# Apical bleeding: a rare cause of blindness after orbital decompression

**DOI:** 10.5935/0004-2749.2022-0006

**Published:** 2023-03-20

**Authors:** Bruna Sâmara Nogueira Equitério, Fabiana Caetano, Hercules Antonio Kozorosky Jr, Antonio Augusto Velasco e Cruz

**Affiliations:** 1 Departamento de Oftalmologia, Faculdade de Medicina de Ribeirão Preto, Universidade de São Paulo, Ribeirão Preto, SP, Brazil; 2 Departamento de Radiologia, Faculdade de Medicina de Ribeirão Preto, Universidade de São Paulo, Ribeirão Preto, SP, Brazil

**Keywords:** Graves ophthalmopathy, Orbital diseases/surgery, Decompression, surgical, Blindness, Humans, Case reports, Oftalmopatia de Graves, Doenças orbitárias/cirurgia, Descompressão cirúrgica, Cegueira, Humanos, Relatos de casos

## Abstract

Orbital decompression is widely performed for the management of proptosis for cosmetic and functional cases of Graves orbitopathy. The main side effects include dry eye, diplopia, and numbness. Blindness after orbital decompression is extremely rare. The mechanisms of vision loss after decompression are not well described in the literature. Considering the devastating effect and rarity of this complication, this study presented two cases of blindness after orbital decompression. In both cases, vision loss was provoked by slight bleeding in the orbital apex.

## Abstract (pt)

A descompressão orbitária é uma cirurgia amplamente empregada para correção da proptose em casos cosméticos e funcionais da orbitopatia de Graves. Os principais efeitos colaterais induzidos pela descompressão são olho seco, diplopia e parestesias. Amaurose pós descompressão é uma complicação extremamente rara e cujos mecanismos são pouco discutidos na literatura. Considerando o efeito devastador representado pela perda visual e a escassez de relatos dessa complicação, os autores apresentam dois relatos de amaurose após descompressão orbitária. Nos dois casos a perda visual ocorreu devido a sangramento de pequena monta no ápice orbitário.

## INTRODUCTION

Blindness is the most feared complication of orbital surgery. This devastating complication very rarely occurs following orbital decompression and is not even mentioned in large retrospective reviews about blindness after orbital surgery^(1,2,3,4)^.

In this study, we present two cases of blindness following orbital decompression resulting from apical bleeding. The procedures were performed by a training surgeon. To the best of our knowledge, this complication has never been reported before.

This study observes the tenets of the Declaration of Helsinki and Health Insurance Portability and Accountability Act regulations.

## CASE REPORTS

### Case 1

A 64-year-old white female patient, with smoking habits and postiodine hypothyroidism, presented with eye exposure and diplopia. She had no coagulopathy and was taking tapazole and propranolol. Examination disclosed proptosis in both eyes (OU, from the Latin *oculus uterque;* Hertel 26/24 mm), limited eye movement in upgaze, and lower eyelid retraction (margin reflex distance 2 [MRD_2_] = 8 mm OU). Her visual acuity was 20/20 in the right eye (OD, *oculus dexter*) and 20/60 in the left eye (OS, *oculus sinister*). She was scheduled for balanced right orbital decompression. The patient asked for decompression for cosmetic reasons only. Radiotherapy was not previously prescribed because her disease was not in the active phase.

The deep lateral wall was decompressed first. When the removal of the medial wall was attempted by transconjunctival incision, the medial rectus was damaged, provoking profuse bleeding along the medial wall. The bleeding was controlled, and the procedure was discontinued. No pupil abnormality was detected during surgery.

**Figure 1:**
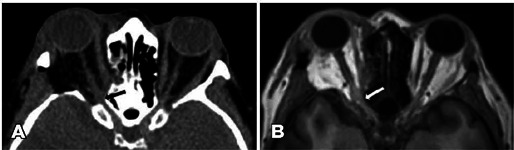
Enlargement of the right optic nerve at the entrance of the optic canal on axial computed tomography (A) and T1-weighted magnetic resonance imaging (B). Globe tenting is absent. Signs of an attempt to decompress the medial wall are also evident.

On postoperative day 1, she reported no light perception in the OD. Computed tomography (CT) and magnetic resonance imaging (MRI) performed on the same day revealed edema of the medial rectus muscle and apical hemorrhage involving the optic nerve canal (Figure 1). Vision restoration by enlarging the medial decompression was unsuccessful. After 2 years of follow-up, permanent right eye blindness ensued.

### Case 2

**Figure 2:**
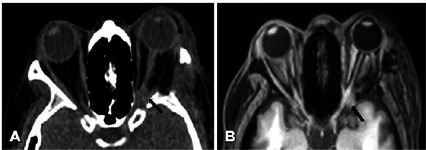
Left deep lateral orbital decompression. The orbital apex is surrounded by blood. The left globe has a normal shape. No signs of increased intraorbital pressure were found.

A 38-year-old woman, who tested positive for human immunodeficiency virus, developed thyroid eye disease with left eye proptosis (Hertel 14/18 mm) and upper eyelid retraction (MRD_1_ = 5 mm OU). Her visual acuity was 20/20 OU. She underwent a deep left lateral orbital decompression. After the procedure, slight bleeding was noted from a temporalis muscle; hence, a Penrose drain was inserted into the lateral space of the orbit. The patient had no history of coagulopathy and was not taking medications that could interfere with blood clotting. On postoperative day 1, she only had light perception on the operated eye. CT and MRI revealed a lateral orbital hemorrhage compressing the optic nerve at the optic canal (Figure 2). During the 1.5-year follow-up, her visual acuity never recovered despite the initiation of intravenous steroid therapy immediately after surgery.

## DISCUSSION

The literature on blindness after orbital decompression comprises only a few case reports^(5,6,7,8,9,10,11)^, and the causative factors associated with vision loss are not well understood. Long and Ellis were probably the first to describe in detail four cases of optic nerve atrophy after lateral decompression. These procedures commonly caused significant bleeding, which were managed with temporary tarsorrhaphy or compressive dressings during the postoperative period. Although none of the patients were examined immediately after surgery, blindness was thought to result from increased intraorbital pressure^(5)^. Ueland also described orbital compartment syndrome as the mechanism of vision loss in a case of unilateral vision loss after a deep lateral decompression complicated by orbital hemorrhage^(6)^. Sellari-Franceschini reported three cases of unilateral vision loss after transantral decompression caused by direct injury to the optic nerve^(7)^. Cruz also reported a case of damage to the optic nerve after cauterization of the posterior ethmoidal neurovascular bundle in a series of 45 three-wall orbital decompressions by a coronal approach^(8)^. Finally, DeSanto reported two cases of blindness following inferomedial decompression, but they did not discuss the possible causative factors involved in the vision loss^(9)^.

To our knowledge, no study has reported vision loss caused by apical bleeding after orbital decompression, wherein a small volume of blood compresses the optic nerve on its entrance in the optic canal. With both MRI and CT, the blood present in the orbital apex caused blurring of the orbital fat between the optic nerve and the muscular cone. CT can distinguish blood from edema because blood has higher Hounsfield units (mean = 60) than fluid (mean = 0)^(12)^.

We believe that meticulous control of any intraconal bleeding is the best way to prevent fatal consequences of apical bleeding. In the cases reported herein, the placement of a Penrose drain, neuroprotection with corticosteroid therapy, and a surgical reapproach did not result in vision recovery.
